# Antimicrobial and Metal Resistance Genes in Bacteria Isolated from Mine Water in Austria

**DOI:** 10.3390/antibiotics14030262

**Published:** 2025-03-04

**Authors:** Jakob Prochaska, Heinz Reitner, Christian Benold, Alfred Stadtschnitzer, Buyantogtokh Choijilsuren, Dmitrij Sofka, Friederike Hilbert, Cátia Pacífico

**Affiliations:** 1 Centre of Food Science and Veterinary Public Health, University of Veterinary Medicine, 1210 Vienna, Austria; jp.tierarzt@gmail.com (J.P.); buyantogtokh.choijilsuren@gmail.com (B.C.); dimitrij.sofka@vetmeduni.ac.at (D.S.); 2 Department of Mineral Resources and Geoenergy, Geosphere Austria, 1030 Vienna, Austria; heinz.reitner@geosphere.at; 3 Department of Geochemistry, Geosphere Austria, 1030 Vienna, Austria; christian.benold@geosphere.at; 4 VA Erzberg GmbH, 8790 Eisenerz, Austria; alfred.stadtschnitzer@vaerzberg.at; 5 Biome Diagnostics GmbH, 1200 Vienna, Austria

**Keywords:** metal resistance genes, antimicrobial resistance, ß-lactamase genes, multidrug efflux pumps, environmental bacteria, species identification

## Abstract

**Background/Objectives:** Microbiomes surrounding mining sites have been found to harbor both antibiotic resistance genes and metal resistance genes. Within the “One Health” framework, which spans human, veterinary and environmental health, it is crucial to determine whether bacterial metal resistance (MR) genes can independently trigger antimicrobial resistance (AMR) or if they are linked to AMR genes and co-transferred horizontally. **Methods and Results**: Bacteria were isolated from an active and an inactive mining site in the alpine region of Austria. Most of the isolated bacteria harbored antimicrobial and metal resistance genes (88%). MALDI-TOF and whole genome sequencing (WGS) revealed that species from the *Pseudomonadaceae* family were the most identified, accounting for 32.5%. All *Pseudomonas* spp. carried AMR genes from the *mex* family, which encode multidrug efflux pumps. β-lactamase production encoded by *bla* genes were detected as the second most common (26%). The same AMR genes have often been detected within a particular bacterial genus. No tetracycline resistance gene has been identified. Among metal resistance genes, *rufB* (tellurium resistance) was the most prevalent (33%), followed by *recGM* (selenium resistance, 30%), *copA* (copper resistance, 26%), and *mgtA* (magnesium and cobalt resistance, 26%). Notably, the *mer* gene family (mercury resistance) was found exclusively in isolates from the inactive mining site (*n* = 6). In addition, genes associated with both antimicrobial and metal resistance, including *arsBM*, *acrD*, and the *mer* operon, were identified in 19 out of the 43 isolates. **Conclusions**: Bacteria isolated from mine water harbored both MR and AMR genes. Given the exceptional diversity of bacterial species in these settings, 16S rRNA gene sequence analysis is the recommended method for accurate species identification. Moreover, the presence of multi-drug transporters and transferable resistance genes against critically important antimicrobials such as fluoroquinolones and colistin identified in these environmental bacteria emphasizes the importance of retrieving environmental data within the “One Health” framework.

## 1. Introduction

Mine water often contains elevated levels of heavy metals and other toxic compounds, creating an extreme environment for microbes. Some bacteria have adapted to these conditions by acquiring metal resistance genes (MR) [1,2,3]. Recent studies suggest that these genes may also contribute to antimicrobial resistance (AMR), either by encoding multidrug efflux pumps or by linking to horizontal gene transfer elements, such as plasmids, integrons, transposons or phages. As a result, AMR and MR genes can be co-transferred, potentially exacerbating the spread of resistant bacteria [4,5,6].

Mining activities in the Alps and in Austria have historically played and continue to make a significant contribution to regional economic development [7]. Austrian mining is particularly focused on the extraction of tungsten, magnesite, talc, iron, and salt, all of which have significant economic value. But global growing demand for minerals used in batteries and electronic devices is expected to increase pressure on mining operations. Abandoned mines and discovered deposits are constantly checked for profitability. This expansion, particularly in metal extraction, poses substantial environmental challenges, including the contamination of important surrounding water systems [7,8].

The “One Health” approach focuses on human health, veterinary health and environmental health, as all domains are interconnected [9]. In human and veterinary health, and to a lesser extent in the environment, antimicrobial substances are used intensively to cure and prevent bacterial disease, leading to increasing antimicrobial resistance in bacteria. There are ancestral genes for AMR in the environment, based on the natural occurrence of antimicrobials produced by bacteria or molds e.g., streptomycin and penicillic acid. Metal resistance genes can be detected in bacteria present in environments with a high metal concentration, be it natural or man-made. The presence of AMR and MR bacteria in mine water is of particular interest due to their potential dual impact: posing ecological risks while also offering biotechnological applications. These microorganisms serve as valuable research subjects for understanding microbial survival mechanisms and their broader ecological implications [10,11]. On the one hand, the resistance traits of these bacteria can facilitate the horizontal transfer of resistance genes and thus pose a threat to human and animal health. On the other hand, their unique metabolic capabilities offer opportunities for bioremediation, biomining, and other environmental applications. Monitoring AMR in environmental bacteria is just as crucial within the “One Health” framework as tracking specific resistant pathogenic clones. Furthermore, the identification of ancestral resistance genes is of the utmost importance, as these may evolve into horizontally transferable genes and thus contribute to the emergence of resistance in pathogenic bacteria and the spread to humans and animals [12,13]. Thus, understanding the co-resistance of AMR and MR, as well as their transmission, is crucial for developing effective strategies to combat AMR, especially since increased mining of certain metals may undermine current approaches.

This study explores the diversity and resistance profiles of bacteria isolated from mine water in Austria, focusing on identifying genes that enable these bacteria to tolerate and adapt to varying metal concentrations and antimicrobial agents. By analysing their genetic and, to some extent, phenotypic properties, we aim to improve our understanding of their ecological roles, their resistance mechanisms, and the possible application in sustainable mining and environmental management. Water samples were collected from two nearby sites, an active iron mine and an abandoned lead and silver mine, offering new insights into the local microbial ecology of mine waters. This research characterizes mechanisms of microbial adaptation to extreme environments and examines the “One Health” implications by exploring the potential co-existence of AMR and MR genes in these bacteria.

## 2. Results

Bacteria were isolated from water and swab samples collected at various locations in two mines. The bacterial colonies were identified using MALDI-TOF species analysis (Supplemental Table S1). Most of the identified species belonged to the *Pseudomonadaceae* family (32.5%), which is most commonly found in aquatic environments. *Pseudomonas* spp. are known for their large genomes, diverse enzymatic activities, and chemotactic abilities, making them valuable for breaking down toxic substances in wastewater and mine waste dumps. In addition to various *Pseudomonas* species, a mixture of Gram-positive and Gram-negative soil bacteria was isolated, including both spore-forming and potentially pathogenic strains (Supplemental Table S1). While 16S rRNA gene sequence analysis provided results that did not agree with MALDI-TOF species identification, there was greater agreement at the genus level. However, some discrepancies were observed, with a mismatch rate of 9%. MALDI-TOF failed to identify the species of 14% of the isolates. These isolates were successfully identified as *Psychrobacillus lasiicapitis*, *Oerskovia turbata*, *Sporosarcina aquimarina*, and *Pedobacter antarcticus* using 16S rRNA gene sequence analysis. In addition, some *Pseudomonas* spp., including *Pseudomonas silesiensis*, *Pseudomonas kielensis* and *Pseudomonas helleri*, showed a poor identification with MALDI-TOF. By using 16S rRNA gene sequence analysis, a single isolate with a lower identity than 97% was predicted as *Bacillus suaedae* with a sequence identity of 96.18% (Supplemental Table S1).

The in silico identification of antibiotic resistance genes was conducted using ABRicate, which enables bulk screening of contigs utilizing seven different databases. The results using the NCBI, CARD, ResFinder, ARG-ANNOT and MEGARES databases can be found in Table 1.

Most resistance genes were identified using MEGARES, including intrinsic resistance genes known for certain species, such as *mex* genes in *Pseudomonas* spp. Some genes, including *cmlV*, *bla*_FOX_, *fosB*, *blaZ*, *oqxB*, and *mrc9*, were consistently detected across all AMR databases, conferring resistance to chloramphenicol, beta-lactams, fosfomycin, fluoroquinolones, quinolones and colistin. Other resistance genes, such as various *bla* genes (beta-lactam resistance), *mphM* (macrolide resistance), *cat* (chloramphenicol resistance), *iri* (rifampin resistance), *van* (vancomycin resistance), and *qnrB* (fluoroquinolone and quinolone resistance) were identified by three to four databases. Many of these genes confer resistance to critically important antimicrobials in human medicine, and some (*qnr* genes, *bla* genes, *cat* genes) are known to be horizontally acquired by pathogens.

Among all bacterial isolates, only five did not contain any antimicrobial or metal resistance genes. Five isolates carried only metal resistance genes, while two harbored only antimicrobial resistance genes, without any detected metal resistance genes. Overall, only 12% of the isolates lacked both metal and AMR genes. The most frequently detected resistance mechanism was multi-drug-efflux pumps (44%). Of the six major antimicrobial classes—ß-lactams, sulfonamides, quinolones (including fluoroquinolones), macrolides, aminoglycoside—resistance genes to ß-lactams were the most widely present (26%). Notably, no tetracycline resistance genes were predicted by any of the databases used. Transmissible quinolone resistance genes, *qnrB39* and *qnrB96*, were detected in two isolates, one from each mine, namely *Buttiauxella massiliensis* and *Serratia inhibens*. Antimicrobial resistance genes from the *mex* and *bla* gene families were most frequently detected. Two major groups of multidrug efflux pumps were identified: the Mex family of efflux pumps in *Pseudomonas* spp. and the OqxB efflux pumps in *Serratia* spp. All *Pseudomonas* isolates found in this study were predicted to have at least one *mex* gene, though no *bla* genes were detected in these isolates. Nevertheless, *bla* genes were primarily found in Gram-negative bacteria and in two *Bacillus* spp. isolates. Among the two *Bacillus* isolates, *B. sanguinis* carried the *bla1* gene, whereas *B. wiedmannii* harbored both *bla1* and *blaZ-12* (also referred to as *bla2*). Both *Bacillus* isolates were isolated from the same mine but collected from different sampling sites. The detection of resistance genes was highly dependent on the species/family. For example, *Serratia* spp. (harboring *oqxB*) and *Buttiauxella massiliensis* (harboring *crp*, *h-ns*, *mcr-9*, *acrB*, *baeR*, *emrR*, *marA*) were isolated from both mines and shared the same AMR genes.

Most isolates were predicted to carry metal resistance genes, with only seven isolates showing no metal resistance genes detected in silico. Notably, all of these bacteria were isolated from the inactive mine, and five of these did not harbor any antibiotic resistance genes either. The number of metal resistance genes per isolate varied, ranging from 1 to 23, though the majority contained 7 or more genes.

**Figure 1:**
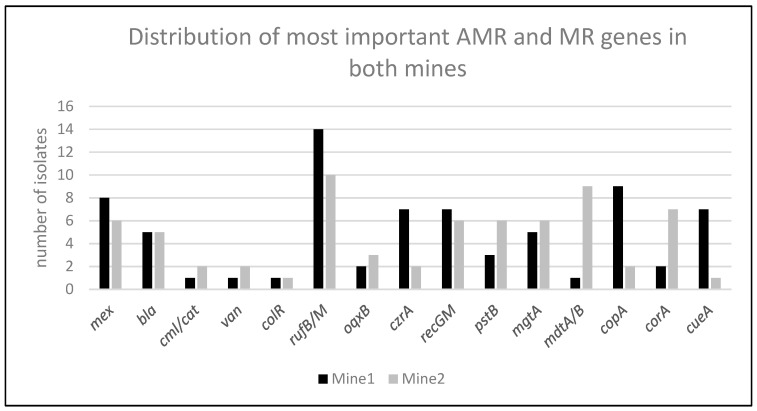
Distribution of most important AMR and MR genes in both mines. For the key antimicrobial resistance genes, no significant difference in their occurrence was observed, as the numbers were too low, and the influence of species was too high to calculate a meaningful statistical correlation. The distribution of the most common metal resistance genes was either equally or unevenly spread in isolates from both mines.

The most frequently predicted metal resistance gene was *ruvB* (tellurium resistance, *n* = 17), followed by *copA* (copper resistance, *n* = 13), *recGM* (selenium resistance, *n* = 12), and *mgtA* (magnesium and cobalt resistance, *n* = 11). While *ruvB*, *recGM*, and *mgtA* were detected in isolates from both mines, *copA* was more commonly found in isolates from mine 1 (Figure 1) [14,15]. Identical MR genes were predominantly observed in specific species. For example, the *recGM* gene, which confers selenium resistance, was found in 12 isolates, all of which were *Pseudomonas* spp., with one *Haemophilus piscium* and one *Aeromonas salmonicida* isolate also carrying the gene. The *acn* gene, associated with copper resistance, was detected in ten isolates, six of which were *Pseudomonas* species, while the remaining four isolates were identified as *Nocardia coeliaca*, *Erwinia rhapontici* and two *Streptomyces venezuelae* isolates. The *mdtB*, responsible for resistance to multiple metals, was found in eight isolates, five of which were *Serratia* spp., with one isolate each of *Pseudomonas gessardi*, *rhapontici* and *Buttiauxella massiliensis*. Additional MR genes, including *ruvB*, were identified across various species.

Predicted resistance genes spanned a wide range of metals, including aluminum, antimony, arsenic, cadmium, cobalt, copper, chromium, iron, lead, mercury, magnesium, manganese, molybdenum, nickel, silver, sodium, sulfur, tellurium, and zinc. Additionally, several multi-resistance genes were identified, such as *arsBM*, *cnrA*, *corA*, *czcP*, *czrA*, *irlR*, *mdtB*, *mdtC*, *mntH*, *nccA*, and *ruvB*. Some genes were associated with both antimicrobial and metal resistance, including *arsBM*, *acrD*, and the *mer* operon. Interestingly, certain genes were exclusive to one mine—for instance, *mer* genes encoding mercury resistance were only detected in the inactive mine. A statistically significant correlation was found between the presence of antimicrobial resistance genes and metal resistance genes (*p* < 0.05). However, due to species diversity and dependencies of AMR genes among bacterial species, additional statistical correlations between specific genes and species could not be determined. Consequently, the correlation between AMR and MR genes could not be determined independently of species. Although MR genes were less species-dependent than AMR genes, the limited number of isolates per species prevented a reliable within-species correlation analysis.

## 3. Discussion

Species identification of isolates was performed using both MALD-TOF analysis and 16S rRNA gene sequence analysis. However, there was limited agreement between these two methods, as has been observed in other studies [16]. This discrepancy may be due to the fact that the MALDI-TOF library is designed primarily for identifying medically relevant pathogens, rather than environmental bacteria. Therefore, for accurate species identification of environmental bacterial isolates, 16S rRNA gene sequence analysis is the preferred method. Only one isolate analyzed via 16S rRNA gene sequencing fell below the 97% identity threshold for species identification [17]. This isolate was predicted as *Bacillus suaedae* at 96.18% identity, and could potentially represent a novel *Bacillus* species, although additional data is required to confirm this.

Individual resistance genes to metals and antimicrobials are commonly found in environmental bacterial isolates [18,19]. Recent studies have reported the potential for co-selection of MR and AMR genes [20]. This co-selection can occur through co-resistance, where genes located within a mobile genetic element are linked and horizontally transmitted, or through cross-resistance, where a single resistance mechanism provides protection against multiple substances, leading to co-selection. Additionally, common regulation of genes via shared promoters or regulators may promote the co-occurrence of resistance. Metals such as cadmium, copper and zinc are particularly associated with co-selection [21]. This study identified co-resistance genes, including *arsBM*, *acrD*, and the *mer* operon. However, evaluating whether specific AMR and MR genes are directly linked was not feasible due to the strong species-dependency observed for AMR genes and the limited number of isolates per species. Although there was a general association between the presence (or absence) of AMR and MR genes in isolates, no direct connection between particular AMR and MR genes could be established.

Antimicrobial resistance genes from the *mex* and *bla* gene families were most frequently detected. No isolate that harbored one gene of the *mex* gene family harbored any *bla* gene, whereas in clinical *Pseudomonas aeruginosa* isolates these genes could be detected simultaneously [22]. Interestingly, no tetracycline, sulfonamide or aminoglycoside resistance genes were identified, although these were commonly found in other studies [23].

Identical MR genes were predominantly seen in specific species, although this species-specific pattern was less pronounced than that observed for AMR genes. For example, the *recGM* gene, which confers selenium resistance, was found in twelve isolates, all of which were *Pseudomonas* spp., with one *Haemophilus piscium* and one *Aeromonas salmonicida* isolate also carrying the gene. The *acn* gene, associated with copper resistance, was detected in ten isolates, six of which were *Pseudomonas* species, while the remaining four isolates were identified as *Nocardia coeliaca*, *Erwinia rhapontici* and *Streptomyces venezuelae*. The *mdtB*, responsible for resistance to multiple metals, was found in eight isolates, five of which were *Serratia* spp., with one isolate each of *Pseudomonas gessardi*, *rhapontici* and *Buttiauxella massiliensis*. These observations highlight the significant influence of species/families on the frequency of detection of resistance genes. This makes it challenging to independently define and impossible to statistically calculate the simultaneous occurrence of antimicrobial and metal resistance genes without considering species or family [24]. However, the horizontal exchange of mobile genetic elements such as plasmids, transposons, integrons or phages is more likely to occur within a family or within closely related families or species than between different families [25]. Further investigation into mobile genetic elements and their association with resistance genes could provide more insight into the sharing of both AMR and MR genes [26,27]. The extensive species diversity in mine waters, along with the species-specific occurrence of AMR, and to a lesser extent, MR genes, significantly limited our ability to calculate correlations between these resistance determinants. While selective plating or enrichment could have increased the number of isolates for specific species or genera, thereby enabling more robust statistical analysis, this approach would have introduced a bias. Instead, our study focused on unselectively isolating bacteria to capture a comprehensive overview of the microbial diversity in this environment and to characterize their AMR and MR gene profiles. In general, most isolates contained more than one resistance gene. Only five isolates (*Pedobacter antarcticus*, *Sporosarcina aquimarina*, *Rossellomorea marisflavi*, *Flavobacterium pectinovorum*, *Psychrobacillus lasiicapiti*) did not harbor either antimicrobial or metal resistance genes. Thus, isolates carrying AMR genes are likely to also contain MR genes in their genomes. Notably, a couple of metal resistance genes were detected in a single isolate, with only 7% of isolates containing only a single resistance gene, confirming other studies [28]. The most frequently detected metal resistance genes were those for copper, zinc, and cadmium, while iron and silver resistance genes were less commonly identified, despite the fact that these isolates were from iron mining and former silver and lead mining sites.

## 4. Materials and Methods

Sample collection. Samples were collected from one active and one inactive mining site in Austria.

1. Mine 1: This site is a former lead and silver mining location. Zinc-containing ore was mined extensively from the 18th century until 1927, when the mine was closed. The old mine is now used as a tourist attraction and for cheese ripening. Four different sampling sites were selected: three water-containing sites in the old deep mining sites and one in the cheese ripening tunnel.
2. Mine 2: This site is known for iron mining. Five sampling locations were chosen, including water from former deep mining areas, metal-containing rock, and two different sludge water sites.

Samples were properly packaged, immediately cooled and transported to the bacteriological laboratory for further analysis.

Isolation of bacteria. Water samples were concentrated through centrifugation and/or filtered and then streaked onto two different types of media. These were one substrate-rich LB agar (Merck KGaA, Darmstadt, Germany) and one poor minimal M63 medium (United States Biological, Salem, MA, USA) with glucose (Merck Millipore, Burlington, VT, USA) as a carbon source. Bacteria were incubated at two different temperatures (20 °C and 10 °C). Single colonies—one to five from each plate grown with colonies—were selected based on their morphology, cultured, and stored in glycerin broth (MH broth: Oxoid, Altrincham, UK; Glycerin: Carl Roth, Karlsruhe, Germany) at −80 °C for future analysis.

Species analysis. Analysis was performed using MALDI-TOF with the MALDI Biotyper^®^ sirius (Bruker Daltonik GmbH, Bremen, Germany). To maximize identification rates, two different sample preparation methods were employed. For the Direct Transfer (eDT) method, a small amount of sample was transferred directly onto a spot on the MALDI target plate using a sterile toothpick or loop. The spot was then overlaid with 1 µL of matrix solution (α-Cyano-4-hydroxycinnamic acid [HCCA], 10 mg/mL in 50% acetonitrile/2.5% trifluoroacetic acid) (Bruker Daltonik GmbH, Bremen, Germany) and allowed to air dry at room temperature. The formic acid extraction method was used when complete cell lysis was necessary, particularly for difficult-to-identify microorganisms. In this method, a small amount of the sample (bacterial colony) was suspended in 300 µL of distilled water, followed by the addition of 900 µL of ethanol (Chem-Lab, Zedelgem, Belgium). The mixture was then centrifuged at 17,000× *g*. The resulting pellet was resuspended in 30 µL of 70% formic acid (Merck, Darmstadt, Germany) and subsequently mixed with 30 µL of acetonitrile (Chem-Lab, Zedelgem, Belgium). After a brief centrifugation, 1 µL of the supernatant was transferred onto the MALDI target plate, overlaid with 1 µL of HCCA matrix solution (Bruker Daltonik GmbH, Bremen, Germany), and allowed to dry. Mass spectra were obtained in positive linear ion mode using the MALDI Biotyper^®^ sirius system (Bruker Daltonik GmbH, Bremen, Germany). Each spectrum was generated by accumulating at least 240 laser shots per spot. Calibration was done using the Bruker Bacterial Test Standard (BST) (ATCC 25922, *Escherichia coli*). The acquired spectra were analyzed using MBT Compass software (version 4.1).

DNA extraction and sequencing. Genomic DNA was extracted from colonies grown on LB-Plates (Sigma Aldrich, St. Louis, MO, USA) at 10 or 20 °C using a QIAamp DNA mini kit (Qiagen, Venlo, The Netherlands). Whole Genome Sequencing was performed at the MicrobesNG facility (Birmingham, UK). For library preparation, the Nextera XT Library Prep Kit (Illumina, San Diego, CA, USA) was used with 2 ng of DNA. DNA sequencing was conducted on an Illumina HiSeq 2500 platform using 2 × 250 bp paired-end approach.

Genome assembly, gene prediction and annotation. Illumina genome sequence reads were assessed for quality using FastQC (version 0.11.9) and subsequently trimmed using Trimmomatic (version 0.3), with a sliding window quality of Q15 and length of 20 base pairs. Kraken (version 2) was used to speciate isolates against all bacterial, archaeal, and viral genomes within the RefSeq database up to 1 November 2017. De novo assembled genomes were produced using SPAdes (version 3.13.0) under default conditions, with the inclusion of the careful flag. Assemblies were also built using SKESA (version 2.3.0) under default conditions. Resulting assembled genomes were annotated using Prokka (version 1.11) under default conditions.

- Bacterial identification. The identity of these bacterial species was determined by performing a BLASTn (assessed 19 April 2024) [29] search on the 16S rRNA gene sequence. Whenever this gene was not intact, the largest partial sequence was taken as in input for BLASTn.
- In silico identification of antimicrobial resistance genes. After WGS, the genomes were screened for known antimicrobial resistance genes using ABRicate (version1.0.1) [30], against NCBI (5286 sequences) [31], CARD (2631 sequences) [32], Resfinder (3077 sequences) [33], ARG-ANNOT (2223 sequences) [34], and MEGARES (6635 sequences) [35,36], databases downloaded on 17 April 2024) according to defaults.
- Metal resistance genes. Annotated genomes were analyzed for the presence of several heavy metal resistance genes. Metal resistance genes were identified performing BLASTn (accessed 23 May 2024) between the nucleotide sequences of the coding sequences and the MEGARES 3.0 database (downloaded on 23 May 2024), in the categories “Metals” and “Multi-compound” [35,36].
- Sequence availability. Sequences are publicly available under PRJNA1224714.
- Statistical analysis. The Fisher’s exact test was used to study differences between antimicrobial resistance and metal resistance genes within the isolates.

## 5. Conclusions

This study aimed to identify bacteria from mine water, an environmental niche recently recognized as significant within the “One Health” framework due to the co-occurrence of metal resistance (MR) and antimicrobial resistance (AMR). We showed now that bacteria isolated from mine water most often carried MR and AMR genes. Isolates harboring MR genes are likely to also possess AMR genes, outlining the importance of screening these environmental niches to public health. The diversity of bacterial species (or genera) isolated from water in both active and inactive mines is high, with *Pseudomonas* species being the most frequently identified. MADI-TOF analysis is not recommended for identifying these environmental bacteria, as 16S rRNA gene sequence analysis provides a more reliable species identification. Bacteria isolated from both inactive and active mine waters harbor a wide array of resistance genes to both antimicrobials and metals, including multi-drug transporters and transferable resistance genes against critically important antimicrobials such as fluoroquinolones and colistin. This underlines the importance of environmental data for the “One Health” framework. The diversity of metal resistance genes detected in the genome of these bacteria is extensive. All isolates without resistance genes (neither antimicrobial nor metal resistance) were detected only in the water of the inactive mine. AMR genes were commonly detected within specific bacterial genera. To the best of our knowledge, this is the first study that analyzed, identified and characterized AMR and MR resistance gene sequences in isolated bacteria from the water from an iron and an abandoned lead and silver mine in Austria.

## Figures and Tables

**Table 1:** Antimicrobial resistance and metal resistance genes of sequenced isolates.

Isolate	NCBI	CARD	Resfinder	ARGANNOT	MEGARES	Metal Resistance Genes
027916_Erz42	*-*	*mexF*, *mexK*, *mexQ*	*-*	*-*	*emhC*, *mexE*, *mexF*, *mexK*, *mexQ*	*actP*, *recGM*, *acn*, *fpvA*, *czrA*, *ruvB*, *modB*, *ruvBM*, *ctpC*, *oscA*
027917_Erz81an	*-*	*mexF*, *mexK*, *mexQ*	*-*	*-*	*emhC*, *mexE*, *mexF*, *mexK*, *mexQ*	*acn*, *modB*, *recGM*, *oscA*, *copS*, *actP*, *ruvB*, *czrA*, *ctpC*, *ruvBM*
027918_Erz4110C	*-*	*mexF*, *mexK*, *mexQ*	*-*	*-*	*emhC*, *mexE*, *mexF*, *mexK*, *mexQ*	*mntH*, *nhaB*, *acrD*, *cueO*, *mgtA (2)*, *terC*, *pstC*, *pstB (2)*, *pitA*, *mdtB*, *corA*, *glpF*
027919_Erz51	*-*	*mexF*	*-*	*-*	*emhC*, *mexE*, *mexF*, *ttgB*	*cinA*, *ruvBM*, *mdtC*, *ruvB*, *irlR*
027920_Erz4210C	*-*	*mexF*	*-*	*-*	*emhC*, *mexE*, *mexF*, *ttgB*	*cinA*, *ruvB*, *irlR*, *ruvBM*, *mdtC*
027921_Erz910C	*-*	mexF	*-*	*-*	*emhC*, *mexE*, *mexF*, *ttgB*	*cadR*, *recGM*, *ruvB*, *ctpC*, *fpvA*, *cueA*, *copR*, *cinA*
027922_Erz62	*cmlV*, *cpt*, *vanR*-*O*	*parY*, *cmlV*, *novA*, *vanRO*	*cmlV_1*	(Phe)*cmlV*, (Phe)*cpt_strepv*	*cmlV*, *cpt*, *novA*, *parY*, *vanRO*	*dnaK*, *acn*, *czcP*, *ideR*
027923_Erz66	*cmlV*, *cpt*, *vanR-O*	*parY*, *cmlV*, *novA*, *vanRO*	*cmlV_1*	Phe)*cmlV*, (Phe)*cpt_strep*	*cmlV*, *cpt*, *novA*, *parY*, *vanRO*	*dnaK*, *acn*, *czcP*, *ideR*
027924_Erz102	*-*	*-*	*-*	*-*	*-*	*dnaK*
027925_Arz111	*-*	*-*	*-*	*-*	*-*	*-*
027926_ARZ121	*-*	*mexF*, *CpxR*, *mexW*	*-*	*-*	*cpxAR*, *emhC*, *mexE*, *mexF*, *ttgB*	*czrA*, *arsB*, *recGM*, *czrA*, *copR (3)*, *ruvB*, *acn*, *copA*, *copD*, *cueA*
027927_ARZ123	*blaSGM-1*	*-*	*blaSGM-1*	(Bla)*blaSGM*-1	*blaSGM*	*copA*, *cnrA*, *nccA (3)*, *ruvB*, *cutO*, *actPC*, *cueA*
027928_ARZ131	*blaSGM-1*	*-*	*blaSGM-1*	(Bla)*blaSGM*-1	*blaSGM*	*nccA (3)*, *ruvB*, *cnrA*, *copA*, *cutO*, *actPC*, *cueA*
027929_ARZ152	*-*	*-*	*-*	*-*	*-*	-
027930_ARZ153	*mphM*, *rphC*	*mphM*, *rphB*	*mphM. rphC*	*-*	*mphB*, *rph*	*alu1P*
027931_ARZ154	*-*	*-*	*-*	*-*	*-*	-
027932_ARZ161	*-*	*-*	*-*	*-*	*-*	-
027933_ARZ162	*-*	*mexF*, *mexW*	*-*	*-*	*emhC*, *mexE*, *mexF*, *ttgB*	*ruvB*, *recGM*, *cadR*, *czrA*, *cueA*, *irlR*, *arsB*, *copA*, *acn*, *copR (2)*, *mertT*, *merA*, *merB*, *copS*, *arsBM*, *pstB*, *ctpC*
027934_ARZ163	*-*	*-*	*-*	*-*	*-*	*actP*, *acn*
027935_ARZ171	*-*	*-*	*-*	*-*	*-*	-
027936_Arz172	*catA*10, *rph*	*rphB*	*cat_4*	(Rif)*rphD*, (Phe)*catA*_variant1	*catA*, *rph*	*alu1P*, *merR1*
027937_ARZ201	*-*	*mexF*, *mexQ*	*-*	-	*emhC*, *mexE*, *mexF*, *mexQ*, *ttgB*	*ruvB*, *mgtA*, *arsB*, *recGM*, *acn*, *copR*, *ruvBM*, *actP*, *copA*, *copB*, *czrA*
027938_ARZ204	*-*	*mexF*, *mexK*, *mexW*	*-*	*-*	*emhC*, *mexE*, *mexF*, *mexK*, *ttgB*	*copA*, *copB*, *copR (2)*, *ruvB*, *copD*, *copS*, *recGM*, *mgtA*, *cueA*
027939_ARZ205	*-*	*mexF*, *mexQ*	*-*	*-*	*emhC*, *mexE*, *mexF*, *mexQ*, *ttgB*	*ruvBM*, *ruvB*, *recGM*, *copR*, *ACN*, *czrA*, *copB*, *copA*, *actP*, *arsB*, *mgtA*
027940_ARZ232	*-*	*-*	*-*	*-*	*-*	*mco*
027941_Erz41an	*blaRAHN-1*, *oqxB9*	*crp*, *hns*, *oqxB*	*blaRAHN-1_1*, *oqxB_1*	(Bla)*blaRAHN-1*, (Flq)*OqxBgb*	*cpxAR*, *crp*, *hns*, *rahN*, *sdeB*	*cueO*, *copA*, *acrD (2)*, *mgtA (2)*, *pitA*, *pstB*, *pstA*, *pstS*, *modC*, *mdtB*, *corA*
027942_Erz51an	*oqxB17*	*crp*, *hns*, *oqxB*	*oqxB_1*	(Flq)OqxBgb	*cpxAR*, *crp*, *hns*, *sdeB*	*cueO*, *acrD*, *mntH*, *mgtA (2)*, *terC*, *pitA*, *pstB (2)*, *pstC*, *nhaB*, *mdtB*, *corA*, *zntA*, *glpF*
027943_Erz61an	*blaFOX-2*, *blaOXA-427*, *cphA1*	*blaFOX-2*, *blaOXA-427*, *cphA5*	*blaFOX-2_1*, *blaOXA-427_1*, *cphA1_1*	(Bla)*blaFOX*-2, (Bla)*blaOXA-12*, (Bla)*cphA5*	*cphA*, *blaFOX*, *blaOXA*	*ruvB*, *ruvBM*, *recGM*
027944_Erz71an	*blaFOX-2*, *blaOXA-427*, *cphA5*	*blaFOX-2*, *blaOXA-427*, *cphA5*	*blaFOX-2_1*, *blaOXA-427_1*, *cphA5_1*	(Bla)*blaFOX-2*, (Bla)*blaOXA-12*, (Bla)*cphA5*	*cphA*, *blaFOX*, *blaOXA*	*zipB*, *cusA*, *ruvBM*, *recGM*
027945_Erz91an	*-*	*crp*, *hns*, *emrR*	*-*	-	*crp*, *emrR*, *hns*	*acn*, *sitABCD*, *mdtB*, *mdtC*, *acrD*, *cueO*, *pstB*, *corC*, *fetB*, *corA*
027946_Arz131an	*iri*	*rpoB2*, *iri*	*-*	*(Rif)iri*	*iri*	*dnaK*, *acn*
027947_Arz143an	*bla1*, *fosB*_gen, *satA_Ba*	*bla1*, *fosB*	*fosB1_1*	*(Bla)bla-1*, *(Fcyn)fosBx1*	*bla1*, *fosB*, *sat*	*-*
027948_Arz231an	bciI, bla1, *fosB*_gen, *satA_Ba*, *vanZ-F*	*bla1*, *bciI*, *fosB*, *vanZF*	*blaZ_12*, *fosB1_1*	*(Bla)bla-1*, *(Bla)bla2*, *(Fcyn)fosBx1*, *(Gly)vanZF-Pp*	*bciI*, *bla1*, *blaZ*, *fosB*, *sat*, *vanYF*	*-*
027949_Arz21110Can	*-*	*mexF*, *cpxR. mexW*	*-*	*-*	*cpxAR*, *emhC*, *mexE*, *mexF*, *ttgB*	*pstB*, *rcGM*, *merR*, *merP*, *merC*, *merA*, *arsBM*, *copR*, *copB*, *ruvB*, *ctpC*, *czrA*, *cueA*, *zraR*
027950_Arz2310C	*-*	*mexF*, *mexK*, *mexW*	*-*	*-*	*emhC*, *mexE*, *mexF*, *mexK*, *ttgB*	*ruvB*, *actP1*, *cueA (2)*, *copR*, *recGM*, *merR2*, *merT*, *merP*, *merF*, *merA*, *merD*, *merE*, *czrA*, *copAM*, *can*
027951_Arz13210C	*-*	*mexF*	*-*	*-*	*emhC*, *mexE*, *mexF*	*copR (2)*, *copS*, *copD*, *copAM*, *pbrA*, *merT (2)*, *merA (3)*, *ctpC (2)*, *zraR*, *ruvB*, *czrA*, *mrdH*, *ruvBM*, *merE*, *merD*, *merF (2)*, *merP (2)*, *merR2*, *ctpV*, *merR*, *oscA*
027952_Arz21110C	*-*	*-*	*-*	*-*	*-*	*ctpG*, *merT*
027953_Arz21210C	*-*	*-*	*-*	*-*	*-*	*czcP*
027954_Erz4110Can	*bla-C*, *oqxB19*, *qnrB39*	*crp*, *hns*, *oqxB*	*oqxB_1*, *qnrB39_1*	(Flq)*oqxBgb*	*blaC*, *cpxAR*, *crp*, *hns*, *qnrB*, *sdeB*, *sdeX*, *sdeY*	*znuC*, *sitABCD*, *mgtA*, *baeR*, *mdtC*, *mdtB*, *pstB*, *acrD*, *zntA*, *corA*, *pstA*, *pstC*, *copR*, *FETB*, *copA*, *corC*
027955_Arz18110Can	*bla-C*, *oqxB11*	*crp*, *hns*, *oqxB*	*oqxB_1*	(Flq)*oqxBgb*	*blaC*, *cpxAR*, *crp*, *hns*, *sdeB*, *sdeX*, *sdeY*	*baeR*, *mdtC*, *mdtB*, *pstB (2)*, *acrD*, *ruvB*, *modC*, *corC*, *mgtA*, *corA*, *pstA*, *pstC*
027956_Erz9210Can	*oqxB9*	*crp*, *hns*, *oqxB*	*oqxB_1*	(Flq)*oqxBgb*	*crp*, *hns*, *sdeB*	*mntH*, *nhaB*, *acrD*, *cueO*, *mgtA*, *terC*, *pstC*, *pstB (2)*, *pitA*, *mgtA*, *mdtB*, *corA*, *glpF*
027957_Erz9110Can	*mcr-9.1*	*crp*, *hns*, *mcr-9*, *acrB*, *baeR*, *emrR*, *marA*	*mcr*-9_1	(*bla*) *E. coli*, (Col)*mcr-9.1*	*acrB*, *cpxAR*, *crp*, *emrR*, *hns*, *marA*, *mcr*, *pbp2*	*mdtA*, *mdtB*, *mdtC*, *baeS*, *baeR*, *ACRD*, *arsBM*, *modC*, *corC*, *mgtA*, *pstS*, *pstC*, *corB*, *dsbA*, *corA*
027958_Arz14110Can	*mcr-9.1*, *qnrB96*	*crp*, *hns*, mcr-9, *acrB*, *baeR*, *cpxA*, *emrR*, *marA*, *msbA*	*mcr-9.1*, *qnrB96*	(Col)*mcr-9.1*	*acrB*, *baeR*, *cpxAR*, *crp*, *emrR*, *hns*, *marA*, *mcr*, *msbA*	*acrD (2)*, *baeR*, *baeS*, *mdtC*, *mdtB*, *mdtA*, *cutE*, *cusA*, *arsBM*, *mgtA*, *pstC*, *pstS*, *cueO*, *corA*

## Data Availability

Assembled genomes are available at the NCBI database using the BioProject ID PRJNA1224714.

## Supplementary Materials

The following supporting information can be downloaded at: https://www.mdpi.com/article/10.3390/antibiotics14030262/s1, Table S1: Identification of bacteria using MALDI-TOF and 16S rRNA gene sequence isolated from mines.
