# Supplementary material for: Antimicrobial and Metal Resistance Genes in Bacteria Isolated from Mine Water in Austria
Source: Antibiotics (Basel). 2025 Mar 4;14(3):262. doi: 10.3390/antibiotics14030262 (PMC11939749; doi:10.3390/antibiotics14030262)
Supplement: Supplementary file 1 [file antibiotics-14-00262-s001.zip › antibiotics-3481761-supplementary.pdf]

Supplemental Table S1. Identification of bacteria using MALDI-TOF and 16S rRNA gene sequence isolated from mines.

Isolate	MALDI-TOF		Best BLAST 16S rRNA gene hit	
	Species	Score Value	Species	Identity in %
027916_Erz42	*Pseudomonas gessardi*	2.07	*Pseudomonas gessardi*	99.80
027917_Erz81an	*Pseudomonas gessardi*	2.21	*Pseudomonas gessardi*[1]	99.90
027918_Erz4110C	*Pseudomonas gessardi*	2.04	*Pseudomonas gessardi*	99.80
027919_Erz51	*Pseudomonas fragi*	2.15	*Pseudomonas weihenstephanensis*	99.67
027920_Erz4210C	*Pseudomonas fragi*	2.10	*Pseudomonas weihenstephanensis*	99.67
027921_Erz910C	*Pseudomonas fragi*	1.50	*Pseudomonas helleri*	99.61
027922_Erz62	*Streptomyces zaomyceticus*	1.98	*Streptomyces venezuelae*	99.60
027923_Erz66	*Streptomyces zaomyceticus*	2.02	*Streptomyces venezuelae*	99.60
027924_Erz102	*Arthrobacter*	1.85	*Paenarthrobacter nitroguajacolicus*	99.80
027925_Arz111	no identification	0	*Pedobacter antarcticus*	99.80
027926_ARZ121	*Pseudomonas frederiksbergensis*	2.07	*Pseudomonas silesiensis*[1]	100.00
027927_ARZ123	no identification	0	*Sphingobium limneticum*	99.93
027928_ARZ131	no identification	0	*Sphingobium limneticum*	99.93
027929_ARZ152	no identification	0	*Sporosarcina aquimarina*	98.82
027930_ARZ153	*Bacillus muralis*	2.22	*Peribacillus frigoritolerans*[1]	99.79
027931_ARZ154	*Bacillus marisflavi*	2.11	*Rossellomorea marisflavi*	100.00
027932_ARZ161	*Flavobacterium*	1.97	*Flavobacterium pectinovorum*	98.20
027933_ARZ162	*Pseudomonas frederiksbergensis*	2.04	*Pseudomonas silesiensis*[1]	99.70
027934_ARZ163	no identification	0	*Oerskovia turbata*	99.78
027935_ARZ171	no identification	0	*Psychrobacillus lasiicapitis*	99.85
027936_Arz172	*Bacillus*	1.73	*Peribacillus muralis*[1]	100.00
027937_ARZ201	*Pseudomonas gessardii*	1.85	*Pseudomonas kielensis*	99.28
027938_ARZ204	*Pseudomonas jessenii*	2.33	*Pseudomonas laurysulfativorans*	99.80
027939_ARZ205	*Pseudomonas libanensis*	1.77	*Pseudomonas kielensis*	99.28
027940_ARZ232	*Pseudomonas libanensis*	1.80	*Bacillus suaedae*	96.18
027941_Erz41an	*Rahnella aquatilis*	2.04	*Serratia liquefaciens*	99.02
027942_Erz51an	*Rahnella inusitata*	1.91	*Serratia liquefaciens*[1]	99.25
027943_Erz61an	*Aeromonas bestiarum*	2.12	*Aeromonas salmonicida*[1]	100.00
027944_Erz71an	*Aeromonas eucrenophila*	2.15	*Haemophilus piscium*	99.80
027945_Erz91an	*Erwinia rhapontici*	2.06	*Erwinia rhapontici*[1]	99.91
027946_Arz131an	*Rhodococcus erythropolis*	2.04	*Nocardia coeliaca*	99.47
027947_Arz143an	*Bacillus thuringiensis*	2.06	*Bacillus sanguinis*	99.74
027948_Arz231an	*Bacillus thuringiensis*	2.19	*Bacillus wiedmannii*[1]	100.00

027949_Arz21110Can	*Arthrobacter kerguelensis*	2.20	*Pseudomonas silesiensis*[1]	99.70
027950_Arz2310C	*Pseudomonas*	1.91	*Pseudomonas silesiensis*	100.00
027951_Arz13210C	*Pseudomonas proteolytica*	2.21	*Pseudomomas gessardi*	99.80
027952_Arz21110C	*Pseudomonas frederiksbergensis*	2.25	*Paeniglutamicibacter psychrophenolicus*	98.80
027953_Arz21210C	*Pseudomonas mandelii*	2.30	*Paeniglutamicibacter psychrophenolicus*	98.80
027954_Erz4110Can	*Serratia fonticola/ Serratia plymuthica*	2.05	*Serratia inhibens*[1]	100.00
027955_Arz18110Can	*Serratia fonticola/ Serratia plymuthica*	2.13	*Serratia inhibens*[1]	100.00
027956_Erz9210Can	*Rahnella inusitata*	1.87	*Serratia liquefaciens*[1]	99.25
027957_Erz9110Can	*Buttiauxella gaviniae*	2.23	*Buttiauxella massiliensis*[1]	100.00
027958_Arz14110Can	*Buttiauxella gaviniae*	2.23	*Buttiauxella massiliensis*	99.48

[1]Partial 16S rRNA gene sequence
